# FOXA1 activates NOLC1 transcription through NOTCH pathway to promote cell stemness in lung adenocarcinoma

**DOI:** 10.1002/kjm2.12930

**Published:** 2025-01-10

**Authors:** Ji‐Fa Li, Xiao‐Qiong Bao, Wen‐Wen Yu, Xiang‐Xiang Chen, Yang‐Yang Ni, Yu‐Bo Shi, Jin‐Cong Wang, Yang‐Jie Sun, Ai‐Li Chen, Wei‐Long Zhou, Hua Ye

**Affiliations:** ^1^ Department of Respiratory and Critical Care Medicine of Affiliated Yueqing Hospital Wenzhou Medical University Yueqing China

**Keywords:** FOXA1, lung adenocarcinoma, NOLC1, NOTCH, stemness

## Abstract

Tumor cell stemness plays a pivotal role in generating functional heterogeneity within tumors and is implicated in essential processes such as drug resistance, metastasis, and cell proliferation. Therefore, creating novel tumor diagnostic techniques and therapeutic plans requires a knowledge of the possible processes that preserve the stem cell‐like qualities of cancers. Bioinformatics analysis of NOLC1 expression in lung adenocarcinoma (LUAD) and prediction of its upstream transcription factors and their binding sites were completed. RT‐qPCR detection of NOLC1 and FOXA1 expression, colony formation assay of cell proliferation, Transwell assay of cell invasion, sphere formation assay of cell stemness, western blot detection of CD133, OCT4, GLI1, NOTCH1 and Hes1 expression, CCK‐8 assay of IC_50_ value of cisplatin, and ChIP and dual‐luciferase reporter validation of binding relationship between NOLC1 and FOXA1 were done. NOLC1 expression was elevated in LUAD cells and tissues. Decreased NOLC1 expression inhibited the proliferation and invasive capacity of LUAD cells, prevented LUAD cells from becoming stem cells, and suppressed cisplatin resistance in the cells. Rescue tests demonstrated that NOLC1 activated the NOTCH pathway to increase the stemness of LUAD cells and promoted cisplatin resistance in LUAD cells. The activation of NOLC1 transcription by FOXA1 was validated by bioinformatics prediction and molecular verification, and the FOXA1/NOLC1 axis enhanced the stemness of LUAD cells. Activation of NOLC1 transcription by FOXA1 through NOTCH pathway promoted stemness of LUAD. FOXA1/NOLC1 axis is expected to become a new target for inhibiting stemness of LUAD cells.

## 1 INTRODUCTION

One of the most prevalent forms of lung cancer, lung adenocarcinoma (LUAD), affects 40% of lung cancer patients and has a 5‐year survival rate of less than 5% for advanced patients.^1^, ^2^ The primary causes of cancer patients' poor survival rates include medication resistance,^3^ distant metastases,^4^ invasion,^5^ and recurrence.^6^ It is important to remember that nearly all of the aforementioned processes are thought to include cancer stem cells or CSCs. By preserving stemness, NCAPG2 encourages erlotinib resistance in LUAD cells.^7^ By controlling the stemness of LUAD cells, XPC may contribute to antitumor creation, preventing tumor invasion and metastasis, and enhancing patient prognosis.^8^ As of right now, CSCs are acknowledged as targets for cancer therapy.^9^, ^10^ Nonetheless, several intricate signaling pathways, such as the Wnt route,^11^ Notch pathway,^12^ Hedgehog pathway,^13^ are found in tumors and control the acquisition of stem cell‐like properties by tumor cells. Current stem cell research for LUAD mostly focuses on differentiation treatment approaches, stem cell function investigations, or the characterization of distinctive markers.^14^, ^15^ The process by which LUAD develops and preserves stem cell‐like traits remains largely unknown. The goal of this work was to investigate possible regulators of LUAD cells' stem cell‐like properties.

The phosphorylated protein known as nucleolar and coiled‐body phosphoprotein 1 (NOLC1) is mostly found in the cell nucleus and is made up of N‐ and C‐terminal domains along with a special central repeating motif.^16^ Functional abnormalities in ribosome biogenesis, including mislocalization of nucleolar proteins, aberrant nucleolar formation, and suppression of rRNA transcription, are caused by overexpression of NOLC1.^17^ Scholars, however, cannot agree on how NOLC1 functions in cancer. Although NOLC1 promotes tumor cell proliferation and migration in colorectal cancer^18^ and nasopharyngeal carcinoma,^19^ overexpression of Runx2 reduces NOLC1 expression in clear cell renal cell carcinoma,^20^ hence promoting tumor cell growth and metastasis. In non‐small cell lung cancer, Huang et al. previously found that NOLC1 was the gene most upregulated in multidrug‐resistant A549 cells. Removing NOLC1 increases the sensitivity of the cells to several medications.^21^ It is interesting to note that Chen et al. discovered a strong link between NOLC1 and breast cancer stemness. Important stemness regulators MYC and ALDH have lower protein levels in breast cancer cells when NOLC1 is knocked down, which prevents the cells from forming spheres.^22^ In light of this, our goal was to uncover the mechanism by which NOLC1 affects the stemness of LUAD cells, opening up a new avenue for CSC‐focused treatment.

This work demonstrated that the NOTCH pathway acted as a mediating factor in the promotion of tumor cells' stemness when NOLC1 expression in LUAD was elevated. Additionally, we looked into the upstream regulatory mechanism behind the high expression of NOLC1 and discovered that LUAD‐expressed FOXA1 was a transcription factor that triggered NOLC1 expression. According to our research, focusing on the FOXA1/NOLC1 axis may offer a novel way to regulate the stemness of LUAD cells and undo the effects of CSCs on metastasis, treatment resistance, or recurrence.

## 2 MATERIALS AND METHODS

### 2.1 Bioinformatics

TCGA (https://portal.gdc.cancer.gov/) provided LUAD‐mRNA expression data (Normal: 59, Tumor: 535). To extract differentially expressed (DE)‐mRNAs, a differential analysis of mRNA between the normal group and the tumor group was carried out using the edgeR program (|logFC|>0.585, FDR < 0.05). The TCGA samples' mRNAsi data were taken from the literature.^23^ Target genes were identified by correlation analysis between DEGs and mRNAsi, and GSEA was used to ascertain the target genes' regulation mechanism. Survival analysis of the TCGA database was performed using the Kaplan–Meier Plotter (http://kmplot.com/analysis/index.php?p=background) to analyze the relationship between the target gene, NOLC1, and survival in LUAD patients. Using the KnockTF database, possible transcription factors upstream of NOLC1 were sought after. The link between NOLC1 and FOXA1 expression was examined using Pearson correlation analysis, and possible binding sites for each in JASPAR were predicted.

### 2.2 Cell culture

LUAD cells (95‐D, H1299, and H1975), 293T cells, and human normal bronchial epithelial cells (BEAS‐2B) were acquired from Sunncell Biotechnology (China) and cultivated in a specific growth medium. The cells were cultivated at 37°C and 5% CO_2_ in a cell culture incubator (Thermo Fisher, USA).

### 2.3 Cell transfection

RiboBio (China) created and produced the small interfering RNAs (si‐NOLC, si‐FOXA1) and short hairpin RNA (sh‐FOXA1) that target NOLC1 and FOXA1, the NOLC1 overexpression plasmid (oe‐NOLC1), and the matching controls. To transfect cells, Lipofectamine 2000 (Invitrogen, USA) was utilized. We bought DAPT (10 μM), an inhibitor of the NOTCH pathway, from MCE (USA).

### 2.4 RT‐qPCR

Following the collection of cells from each group, total RNA was extracted using the TRIzol® reagent (Invitrogen, USA). Using a NanoDrop 2000 spectrophotometer (Thermo Fisher, USA) to measure RNA quality and concentration, reverse transcription was carried out using a Bestar qPCR RT Kit (DBI, Germany). RT‐qPCR was carried out using Bestar Sybr Green qPCR Master Mix (DBI, Germany) on a Stratagene Mx3000P real‐time PCR system (Agilent, USA). Using the 2^−∆∆ct^ technique, the relative gene expression levels were determined. Table 1 contains a list of the primers.

**TABLE 1:** RT‐qPCR primers.

Gene	Sequence
NOLC1	
Forward	5′‐AAGAAGCCACAGAAGGTAGCA ‐3′
Reverse	5′‐CACTGGAGTCATCAGAAGAAGAAC ‐3′
FOXA1	
Forward	5′‐AAGGGCATGAAACCAGCGAC ‐3′
Reverse	5′‐GCCTGAGTTCATGTTGCTGAC ‐3′
GAPDH	
Forward	5′‐TGTTCGTCATGGGTGTGAAC ‐3′
Reverse	5′‐ATGGCATGGACTGTGGTCAT ‐3′

### 2.5 Western blot

Prechilled RIPA cell lysis buffer (Beyotime, China) was used to extract the total proteins from the cells. After quantification of protein concentration using a BCA protein assay kit (Beyotime, China), sample concentrations were calibrated for consistency. SDS‐PAGE was used to separate proteins, followed by transferring the separated proteins to a PVDF membrane. After blocking with 5% skim milk for 2 h, the primary antibody was incubated at 4°C for the whole night. The secondary antibody was incubated at room temperature for 1.5 h following TBST washing. Protein blot development was done using an ECL solution (Millipore Sigma, USA). Table 2 contains a list of the necessary antibodies.

**TABLE 2:** Antibody list.

Name	Article number	Experiment	Company
CD133	ab222782	WB	Abcam, UK
OCT4	ab181557	WB	Abcam, UK
Hes1	ab108937	WB	Abcam, UK
NOTCH1	ab52627	WB	Abcam, UK
Gli1	ab134906	WB	Abcam, UK
IgG	ab150077	WB	Abcam, UK
GAPDH	ab9485	WB	Abcam, UK

### 2.6 Colony formation assay

Each group's cells were planted into a 12‐well plate at a density of 400 cells per well, and they were continuously cultivated for 2 weeks. After 30 min of 75% alcohol fixation, cells were stained for 15 min with 0.5% crystal violet. Both the number of colonies and photos were recorded.

### 2.7 Transwell assay

The Transwell chamber method was used to assess the invasive ability of cells. Matrigel matrix gel (BD Biosciences, USA) was diluted in serum‐free cell culture medium to 100 μL and coated on the chamber. They were incubated at 37°C in a culture chamber for 4 h to allow the Matrigel matrix gel to polymerize into a film. Two hundred microliters (1 × 10^4^ cells) of serum‐free culture medium was added to the upper chamber, and 600 μL medium containing 10% FBS was added to the lower chamber. After incubation at 37°C in a culture chamber for 72 h, cells were fixed with 4% paraformaldehyde and stained with crystal violet dye for 30 min. Random fields were captured using a microscope (Olympus, Japan). The number of invading cells was calculated using ImageJ counting software.

### 2.8 Sphere formation assay

Cells from each group were planted in ultralow attachment 6‐well plates (4 × 10^4^ cells/well; Corning, USA). Human EGF (20 ng/mL, Invitrogen, USA), B27 (20 ng/mL, Invitrogen, USA), β‐FGF (10 ng/mL, Invitrogen, USA), and IGF (20 ng/mL, Cell Signaling, USA) were added to serum‐free DMEM (Gibco, USA) to feed the cells throughout growth further. Two weeks later, the number of spheres was tallied.

### 2.9 IC_50_ value detection

After cell collection, cells were seeded at a density of 5000 cells per well into a 96‐well plate. Once the cells adhered, different concentrations of cisplatin (0, 5, 10, 15, 20, and 25 μM) were added. After 24 h, 10 μL per well of CCK‐8 reagent (Beyotime, China) was added, and the plate was incubated in a culture chamber for 3 h. The absorbance at OD 450 nm was measured using a microplate reader (BioTek Instruments, USA). The IC_50_ value was calculated using GraphPad Prism 8.0.

### 2.10 ChIP

Around 100 mm cell culture dishes were seeded with 3 × 10^6^ cells. RIPA cell lysate was added after cells were cross‐linked with 1% formaldehyde, and samples were sonicated to extract DNA fragments with an average length of 200–500 bp. After removing the supernatant, anti‐FOXA1 or IgG was incubated for an additional night at 4°C. To precipitate the complex, protein A/G‐Sepharose beads (GE Healthcare, USA) were added and incubated for 4 h. qPCR amplification was performed using the eluted DNA that had precipitated. Table 3 contains a list of primers.

**TABLE 3:** ChIP qPCR primers.

	Sequence
Site	
Forward	5′‐AGGACCTAGAGAAGCTCACACT ‐3′
Reverse	5′‐GTGCCATGCAGTGTGCTCAA ‐3′

### 2.11 Dual‐luciferase assay

Magic Biotech (China) designed luciferase reporter gene vectors (PGL3basic) with the wild‐type (WT) or mutant (MUT) NOLC13’‐UTR sequence. Using Lipofectamine 2000 (Invitrogen, USA), NOLC1‐WT or NOLC1‐MUT vector and si‐FOXA1 were co‐transfected into 293 T cells. Using the Dual luciferase Reporter Gene Assay kit (Promega Corporation, USA), luciferase activity was tested after 48 h.

### 2.12 Subcutaneous xenograft mouse model

Nine female C57BL/6 mice (6–8 weeks old, 20–23 g) were purchased from Hangzhou Qizhen Experimental Animal Technology Co., Ltd. and maintained in a specific pathogen‐free (SPF) environment. The animal protocol and experimental procedures were approved by the Ethics Committee of Yueqing People's Hospital and conducted in accordance with the Guidelines for the Care and Use of Laboratory Animals of Yueqing People's Hospital. The mice were randomly divided into 3 groups (*n* = 3) and subcutaneously injected with 1 × 10^6^ H1975 cells transfected with sh‐NC + oe‐NC, sh‐FOXA1 + oe‐NC, and sh‐FOXA1 + oe‐NOLC1, respectively, in the abdominal region. Tumor volumes were measured starting from the 7th‐day postinjection and then weekly using the formula: tumor volume = (length × width^2^)/2. On the 21st day, the mice were euthanized, tumors were excised, and subsequent experiments were carried out.

### 2.13 Statistical analysis

GraphPad 8.0 (GraphPad Software, Inc.) was used to statistically analyze all of the data in this study. The above‐mentioned studies were all run in triplicate and are shown as mean ± standard deviation. Student's t‐test was used to analyze differences between two groups, while one‐way analysis of variance was used to compare differences between several groups. When *p* < 0.05, a difference is deemed statistically significant.

## 3 RESULTS

### 3.1 NOLC1 is highly expressed in LUAD

**FIGURE 1:**
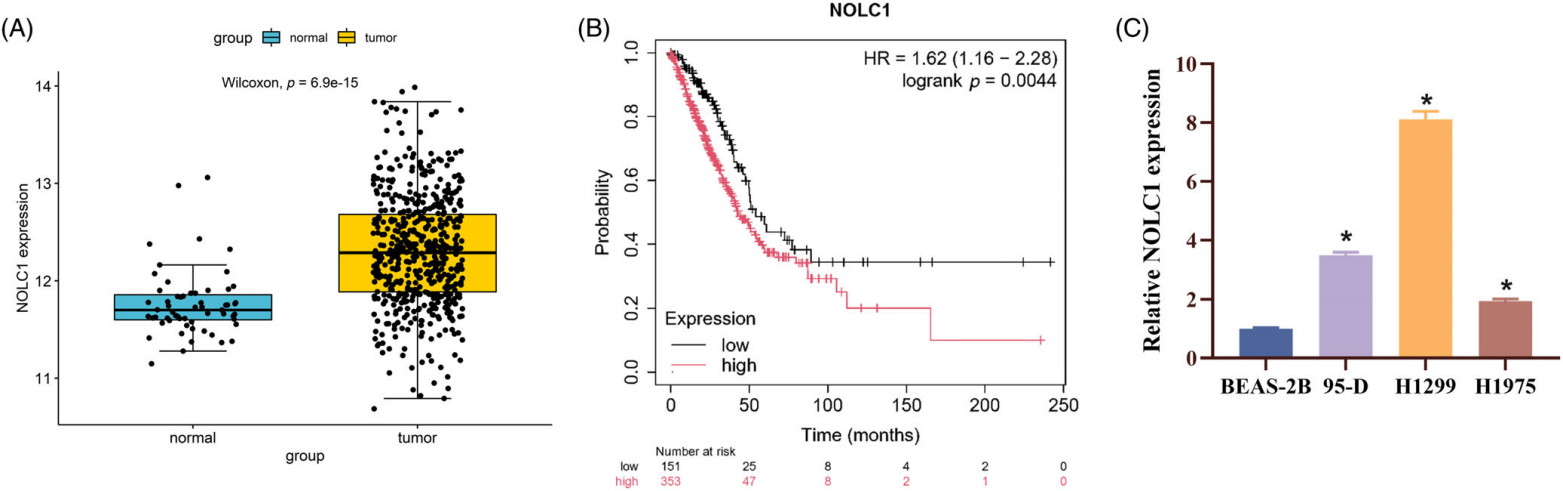
High expression of NOLC1 in LUAD. (A) TCGA analysis of NOLC1 expression in LUAD tissues. (B) Kaplan–Meier curve analysis of survival in LUAD patients with different NOLC1 expression. (C) RT‐qPCR detection of NOLC1 expression in LUAD cells. * indicates *p* < 0.05.

Using the TCGA LUAD mRNA dataset, we ran edgeR differential analysis and discovered that NOLC1 was highly elevated in LUAD (*p* = 6.9e‐15) (Figure 1A). Then significant poor prognosis was found by Kaplan–Meier curve analysis in LUAD patients with high NOLC1 expression (Figure 1B). The expression of NOLC1 was consistently greater in LUAD cells (95‐D, H1299, and H1975) than in human normal bronchial epithelial cells (BEAS‐2B), according to RT‐qPCR data (Figure 1C). In conclusion, LUAD had significant levels of NOLC1 expression.

### 3.2 NOLC1 promotes stemness of LUAD cells

**FIGURE 2:**
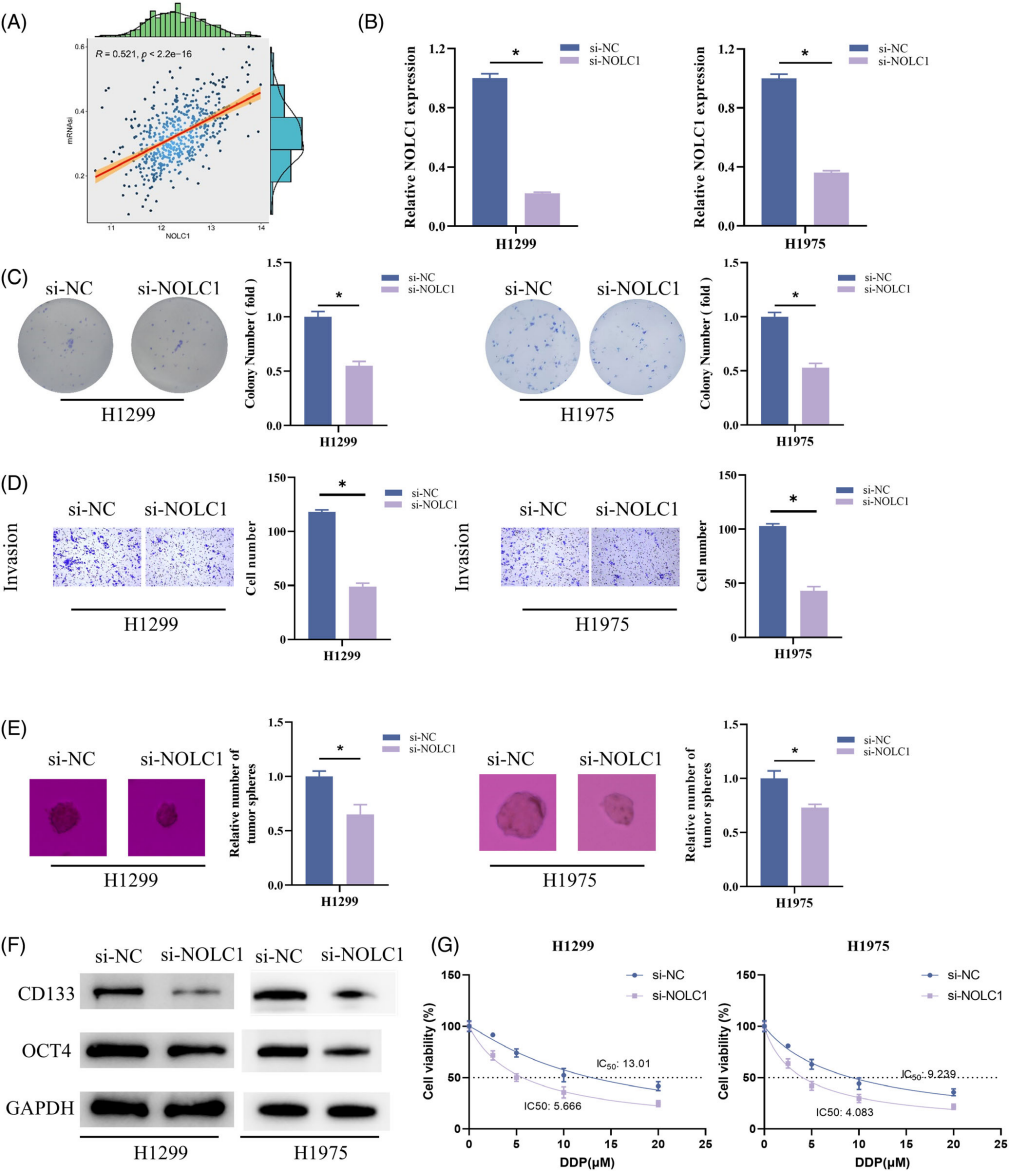
NOLC1 promotes stemness of LUAD cells. (A) Correlation analysis of NOLC1 with mRNAsi. (B) RT‐qPCR detection of NOLC1 expression. (C) Colony formation assay. (D) Cell invasion assay. (E) Sphere formation assay. (F) Western blot detection of CD133 and OCT4 expression. (G) Determining the IC_50_ value of cells to cisplatin using CCK‐8 assay. * indicates *p* < 0.05.

In breast cancer, NOLC1 is substantially enriched in pathways relevant to stem cells.^22^ We sought to find out if NOLC1 in LUAD exhibited any of the same traits. A substantial positive association (*p* < 2.2e‐16) was found in the correlation study between NOLC1 and mRNAsi (Figure 2A). We created H1299 and H1975 cells with much lower NOLC1 expression to confirm the function of NOLC1 in the stemness of LUAD cells (Figure 2B). Experiments on colony formation, cell invasion, and sphere formation showed that knockdown of NOLC1 impeded the ability of cells to proliferate, invade, and form spheres (Figure 2C–E). Furthermore, knocking down NOLC1 decreased the expression of OCT4 and CD133, as demonstrated by WB detection of stem cell markers (Figure 2F). Increased stemness affects the development of cisplatin resistance in cancer.^24^ We found that H1299 and H1975 cells were significantly less resistant to cisplatin after NOLC1 knockdown by IC_50_ value detection (Figure 2G). To sum up, NOLC1 enhanced LUAD cell stemness and cisplatin resistance.

### 3.3 NOLC1 promotes the stemness of LUAD cells through NOTCH pathway

**FIGURE 3:**
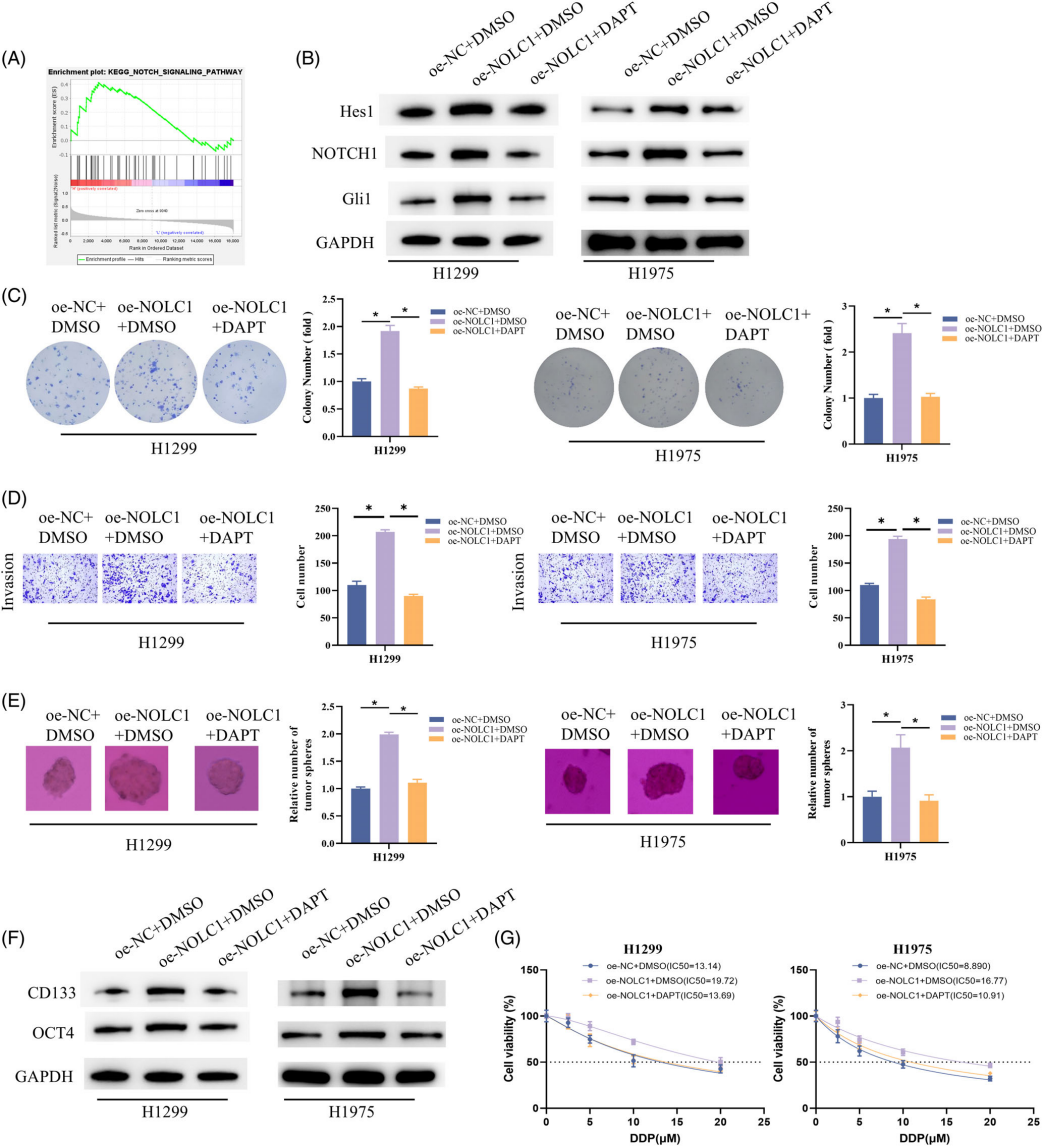
NOLC1 promotes stemness of LUAD cells through NOTCH pathway. (A) GSEA. (B) Western blot detection of Hes1, NOTCH1, and Gli1 expression. (C) Colony formation assay. (D) Cell invasion assay. (E) Sphere formation assay. (F) Western blot detection of D133 and OCT4 expression. (G) Determining the IC_50_ value of cells to cisplatin using CCK‐8 assay. * indicates *p* < 0.05.

After that, we ran GSEA on NOLC1 and discovered a noteworthy NOTCH signaling pathway enrichment (Figure 3A). We administered the γ‐secretase inhibitor DAPT (10 μM), which can decrease NOTCH1 activation, to H1299 and H1975 cells that overexpress NOLC1. According to WB data, overexpression of NOLC1 increased the expression of Hes1, NOTCH1, and Gli1, three important genes in the NOTCH pathway; however, DAPT could reverse this promotion (Figure 3B). As shown by the colony, Transwell, and sphere formation experiments, overexpression of NOLC1 increased H1299 and H1975 cell proliferation, invasion, and sphere‐forming capacity, which could be returned to control levels when DAPT blocked the NOTCH pathway (Figure 3C–E). In the meantime, NOLC1 overexpression's encouraging impact on the production of CD133 and OCT4 proteins was suppressed by DAPT treatment (Figure 3F). Detection of IC_50_ values showed that overexpression of NOLC1 significantly promoted cisplatin resistance in H1299 and H1975 cells but was significantly reduced upon the addition of DAPT treatment (Figure 3G). In conclusion, promotion of stemness and cisplatin resistance in LUAD cells by NOLC1 was mediated through NOTCH pathway.

### 3.4 FOXA1 activates transcription of NOLC1

**FIGURE 4:**
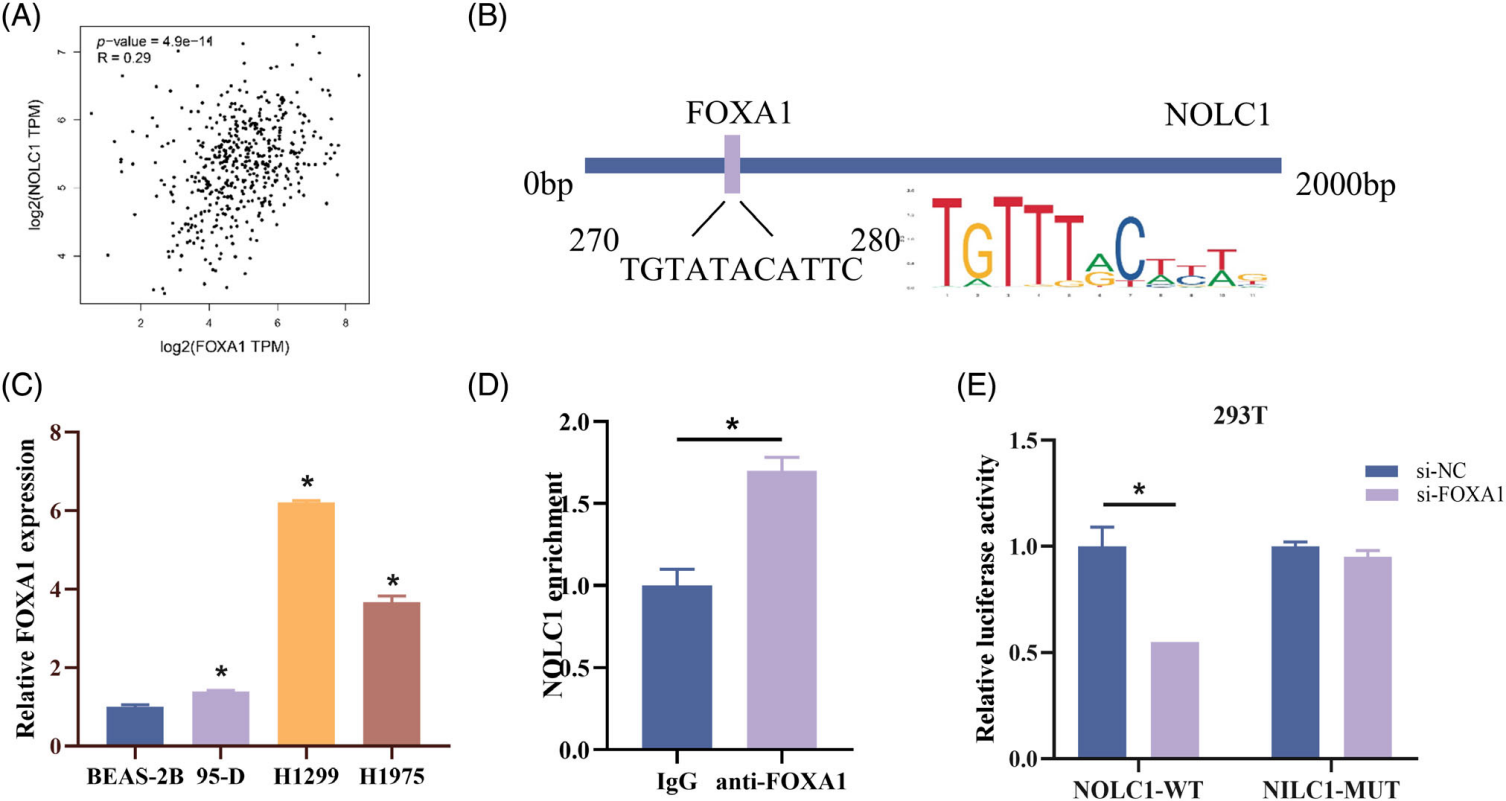
FOXA1 activates NOLC1 transcription. (A) Correlation analysis of FOXA1 and NOLC1. (B) Prediction of binding sites between FOXA1 and NOLC1. (C) RT‐qPCR detection of FOXA1 expression in LUAD. (D) ChIP assay. (E) Dual‐luciferase reporter assay. * indicates *p* < 0.05.

To gain more insight into the regulatory mechanism upstream of NOLC1, we performed correlation analysis with NOLC1 and used the KnockTF database to find putative transcription factors of NOLC1. The results showed a strong positive correlation (*p* = 4.9e‐11) between the expression of NOLC1 and FOXA1 (Figure 4A). Potential binding sites between the two were found using JASPAR prediction (Figure 4B). Later, FOXA1 mRNA expression in cells was found, with LUAD cells exhibiting noticeably greater expression (Figure 4C). We conducted ChIP‐qPCR and dual‐luciferase reporter assays, which verified that FOXA1 can bind to the NOLC1 promoter area (Figure 4D). Furthermore, FOXA1 knockdown reduced the NOLC1‐WT group's luciferase activity, while having no impact on the NOLC1‐MUT group (Figure 4E). In summary, FOXA1 could bind to NOLC1 and start its transcription.

### 3.5 FOXA1 transcriptionally activates NOLC1 to promote stemness in LUAD cells

**FIGURE 5:**
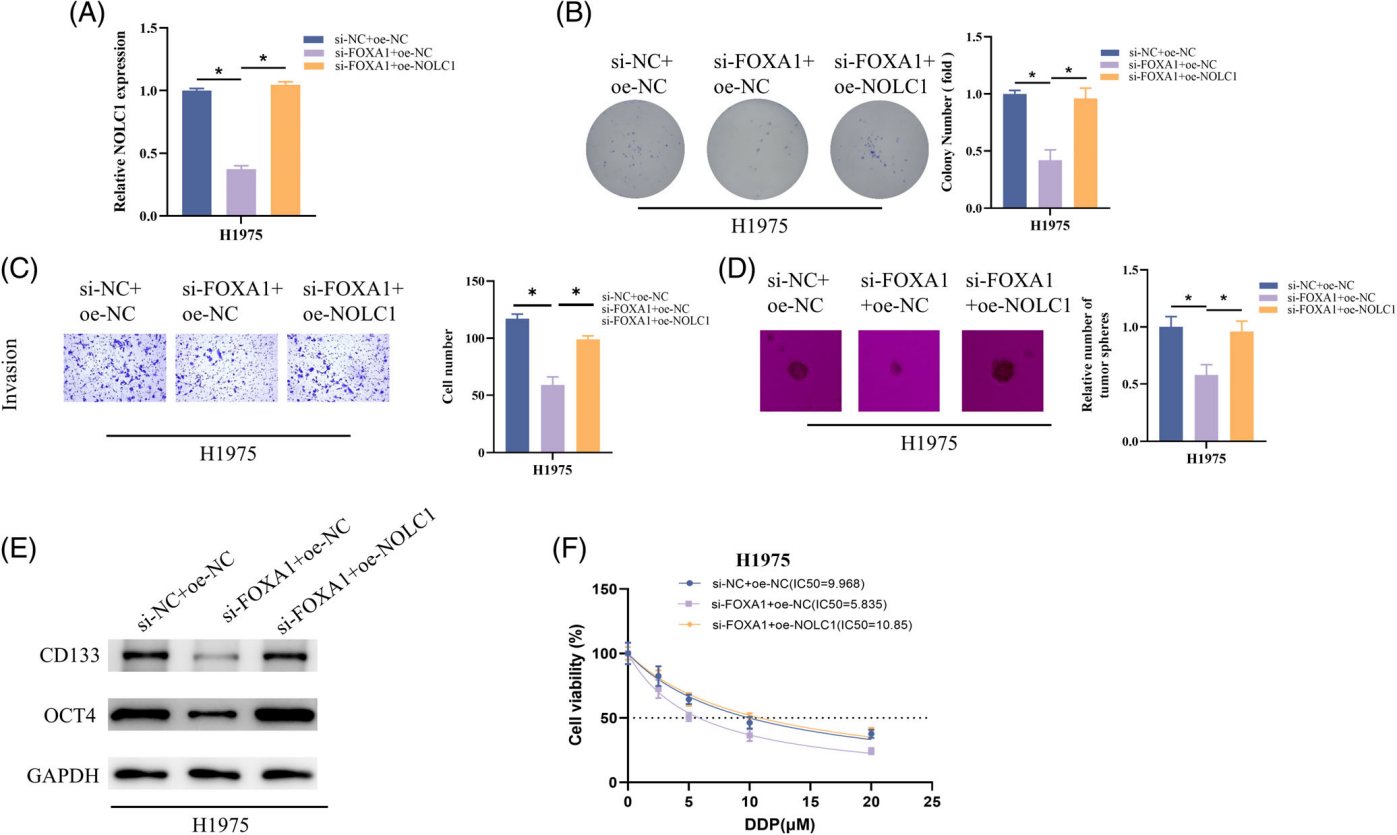
FOXA1 transcriptionally activates NOLC1 to promote stemness of LUAD cells. (A) RT‐qPCR detection of FOXA1 expression. (B) Colony formation assay. (C) Cell invasion assay. (D) Sphere formation assay. (E) Western blot detection of CD133 and OCT4 expression. (F) Determining the IC_50_ value of cells to cisplatin using CCK‐8 assay. * indicates *p* < 0.05.

Lastly, confirmation is required regarding the influence of the FOXA1/NOLC1 axis on stemness in LUAD cells. We produced H1975 cells with either a single FOXA1 knockdown or a simultaneous FOXA1 knockdown and overexpression of NOLC1, together with the appropriate controls. The FOXA1 knockdown group's NOLC1 expression was decreased, but it was later recovered with further overexpression of NOLC1, according to RT‐qPCR analysis (Figure 5A). In the assays involving colony, Transwell, and sphere formation, H1975's capacity to proliferate, invade, and form spheres was decreased by knocking down FOXA1, but this impact was reversed when NOLC1 was overexpressed (Figure 5B–D). When FOXA1 was knocked down, CD133 and OCT4 expression was blocked for western blot detection; however, when NOLC1 was further overexpressed, CD133 and OCT4 expression was restored (Figure 5E). In addition, knockdown of FOXA1 significantly reduced cisplatin resistance in H1975 cells compared to controls but returned to control levels after overexpression of NOLC1 (Figure 5F). In conclusion, FOXA1 transcriptionally activated NOLC1 to drive LUAD cell stemness and cisplatin resistance.

### 3.6 NOLC1 expression promotes tumor growth and enhances the stemness of LUAD in vivo

**FIGURE 6:**
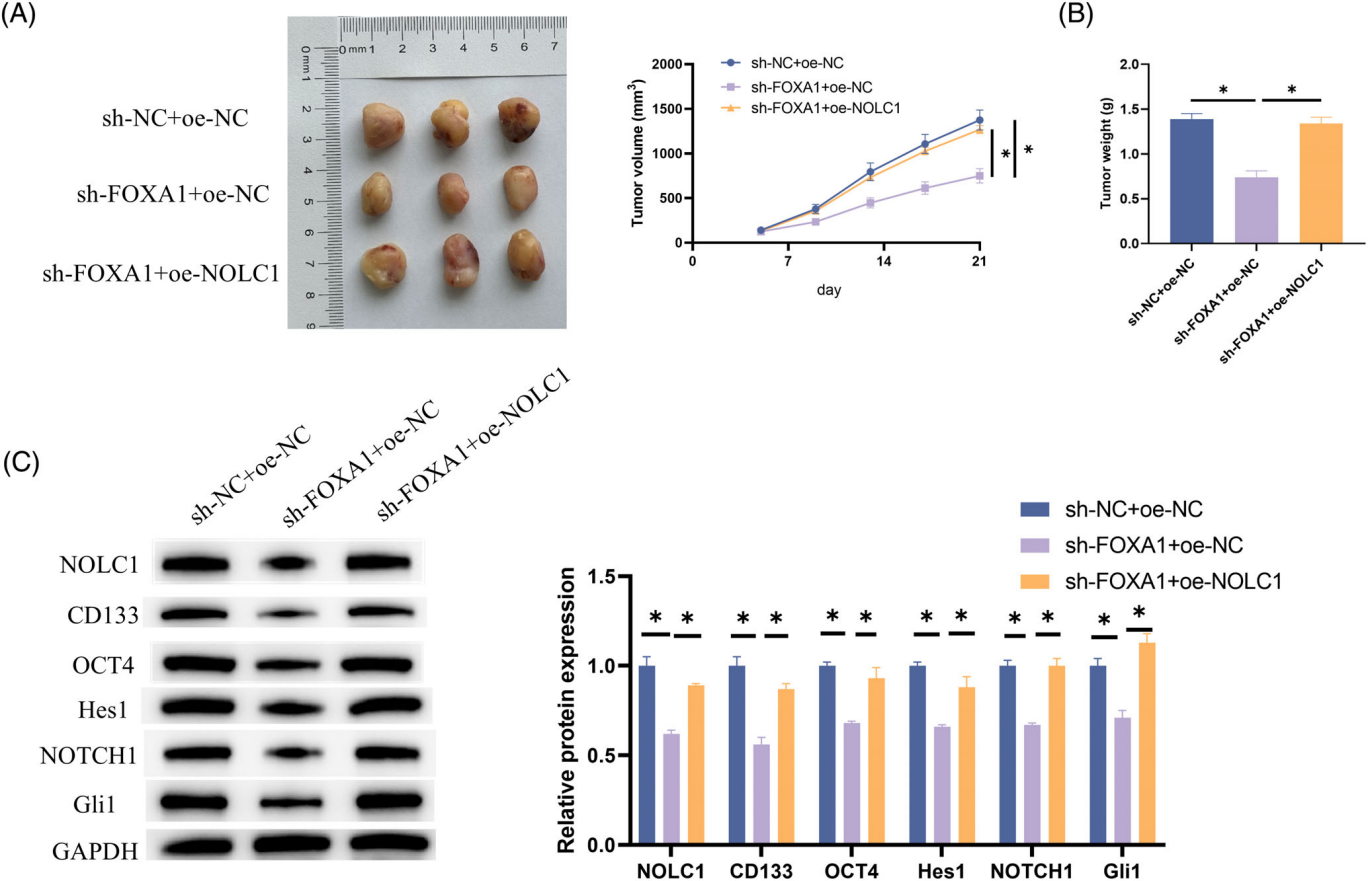
NOLC1 promotes tumor growth and enhances stemness of LUAD in vivo. (A) Growth curve and representative images of LUAD tumors (*n* = 3). (B) Measurement of LUAD tumor weights. (C) Western blot detection of NOLC1, CD133, OCT4, Hes1, NOTCH1, and Gli1 expression. * indicates *p* < 0.05.

To validate the role of NOLC1 in vivo, we established a xenograft mouse model by subcutaneously injecting H1975 cells transfected with sh‐NC + oe‐NC, sh‐FOXA1 + oe‐NC, and sh‐FOXA1 + oe‐NOLC1 into mice. Examination of tumor growth revealed that knockdown of FOXA1 significantly inhibited tumor growth in mice. However, this growth inhibition was notably alleviated when NOLC1 was overexpressed concurrently with FOXA1 knockdown (Figure 6A,B). Subsequently, tumor tissues from the mice were analyzed using western blot to assess the expression changes of NOLC1, stemness markers, and proteins in the NOTCH signaling pathway. It was observed that after FOXA1 knockdown, the expressions of NOLC1, CD133, OCT4, Hes1, NOTCH1, and Gli1 were significantly downregulated, but this effect was mitigated upon NOLC1 overexpression (Figure 6C). In conclusion, in the in vivo environment, FOXA1 regulated NOLC1 expression, thereby promoting the stemness of LUAD.

## 4 DISCUSSION

CSCs are a subset of cancer cells that possess stem cell traits, such as the ability to self‐renew and produce differentiated progeny.^9^ By modifying internal and extrinsic tumor adaptations, CSCs contribute positively to the development of cancer and resistance to therapy.^25^ Several regulatory networks have tight supervision over CSCs.^26^ Employing the Wnt/β‐catenin/c‐Myc/SOX2 axis, TM4SF1 preserves the stemness and EMT of cancer cells during colorectal cancer recurrence and metastasis.^27^ Tumor‐associated fibroblasts, which interact with tumor cells via the OPN/SPP1‐CD44 axis, enhance the stemness of pancreatic cancer cells.^28^ According to our research, NOLC1 expression in LUAD was strongly expressed, which is in line with the pattern of expression in colorectal and prostate cancers.^18^, ^29^ Furthermore, NOLC1 knockdown can prevent LUAD cells from being stem cells. This shows that NOLC1 could be a viable target to reduce LUAD cells' stemness, reverse chemoresistance, and stop LUAD from developing into malignancies.

NOLC1 was considerably enriched in the NOTCH pathway, according to GSEA analysis. The NOTCH system is implicated in several tumorigenic pathways, including inappropriate cell‐to‐cell communication,^30^ metabolic abnormalities,^31^ and tumor microenvironmental control.^32^ Triggering the NOTCH pathway is a complicated process including enzymatic cleavage, heterodimerization, and receptor nuclear translocation.^33^ Numerous studies have examined the NOTCH pathway's stimulation of CSCs in a variety of tumor types; however, because tumor heterogeneity affects the NOTCH pathway's activation mechanism, it differs. While MOGS enhances NOTCH1 protein maturation and activates the NOTCH pathway by boosting NOTCH1 glycosylation, which in turn promotes stemness acquisition and tumor invasiveness in colorectal cancer,^34^ TM4SF1 activates the NOTCH pathway by positively regulating MYH9, which in turn promotes hepatocellular carcinoma stemness and enables it to acquire Lenvatinib resistance.^35^ The rescue experiments we performed demonstrated that the promotion of LUAD stemness by overexpression of NOLC1 could be eliminated by NOTCH inhibitors. We are pleased to find that researchers have demonstrated the antitumor properties of NOTCH inhibitors in a variety of cancer types,^36^ and over the past two decades, successively more than a dozen inhibitors or blocking antibodies specific for NOTCH signaling have been tested in clinical trials or preclinical studies in solid and hematologic malignancies. Our data provide guidance for the application of NOTCH inhibitors in the management of LUAD.

Upon investigating the regulatory genes upstream of NOLC1, it was discovered that FOXA1 was significantly expressed in LUAD and had the ability to trigger the transcription of NOLC1. The protein Forkhead box A1 (FOXA1) belongs to a class of unique transcription factors (TFs) called pioneer factors. Other transcription factors in that area are activated as a result of these pioneer factors' binding to condensed, inactive chromatin, and chromatin remodeling.^37^ When it comes to non‐small cell lung cancer, FOXA1 stimulates the ERK1/2 and JAK2 pathways that are mediated by CDC5L.^38^ It also prevents autophagy‐dependent cell death through the IGF2/IGF1R/mTORC1 signaling pathway, which gives LUAD cells an edge when they are starved of nutrients.^39^ Our cell tests verified that further overexpressing NOLC1 may counteract the inhibitory impact of FOXA1 knockdown on the stemness and drug resistance of LUAD cells. Eliminating stemness in LUAD cells may be accomplished via targeting the FOXA1/NOLC1 axis.

In the present study, we found that FOXA1 promoted cisplatin resistance by activating NOLC1 transcription through an enhanced NOTCH signaling pathway, thus promoting stemness in LUAD cells. This provides a strong complement to the mechanism of NOTCH pathway activation in LUAD, suggesting that targeting the FOXA1/NOLC1 axis is an effective way to regulate LUAD NOTCH pathway and suppress tumor cell stemness. However, previous studies have demonstrated the regulatory role of CSCs in tumor initiation, metastasis, drug resistance, recurrence, etc. The present study only provides preliminary insights into the mechanism of stemness acquisition in LUAD cells and the effect on cisplatin resistance, while it is uncertain whether the FOXA1/NOLC1 axis can regulate other malignant phenotypes by affecting LUAD cell stemness. In addition, the key molecules linking the NOLC1 and NOTCH pathways also remain unclear. We will elucidate these matters in further research to facilitate the creation of precisely tailored treatments for individuals with LUAD.

## CONFLICT OF INTEREST STATEMENT

The authors declare no conflict of interest.

## ETHICS STATEMENT

The animal protocol and experimental procedures were approved by the Ethics Committee of Yueqing People's Hospital and conducted in accordance with the Guidelines for the Care and Use of Laboratory Animals of Yueqing People's Hospital.

## Data Availability

The data and materials in the current study are available from the corresponding author on reasonable request.
